# Identification of Hypericin as a Candidate Repurposed Therapeutic Agent for COVID-19 and Its Potential Anti-SARS-CoV-2 Activity

**DOI:** 10.3389/fmicb.2022.828984

**Published:** 2022-02-10

**Authors:** Aline da Rocha Matos, Braulia Costa Caetano, João Luiz de Almeida Filho, Jéssica Santa Cruz de Carvalho Martins, Michele Gabrielle Pacheco de Oliveira, Thiago das Chagas Sousa, Marco Aurélio Pereira Horta, Marilda Mendonça Siqueira, Jorge Hernandez Fernandez

**Affiliations:** ^1^Laboratório de Virus Respiratórios e do Sarampo, Insituto Oswaldo Cruz, Fundação Oswaldo Cruz (LVRS-IOC-Fiocruz), Rio de Janeiro, Brazil; ^2^Laboratório de Química e Função de Proteínas e Peptídeos, Centro de Biociências e Biotecnologia, Universidade Estadual do Norte Fluminense (LQFPP-CBB-UENF), Campos dos Goytacazes, Brazil; ^3^Plataforma de Laboratórios de Biossegurança Nível 3, Instituto Oswaldo Cruz, Fundação Oswaldo Cruz (NB3-IOC-Fiocruz), Rio de Janeiro, Brazil

**Keywords:** SARS-CoV-2, COVID-19, Hypericin, RdRp, Mpro, drug repurposing

## Abstract

The COVID-19 pandemic has had an unprecedented impact on the global economy and public health. Its etiologic agent, the severe acute respiratory syndrome coronavirus 2 (SARS-CoV-2) is highly transmissible, pathogenic and has a rapid global spread. Currently, the increase in the number of new confirmed cases has been slowed down due to the increase of vaccination in some regions of the world. Still, the rise of new variants has influenced the detection of additional waves of rising cases that some countries have experienced. Since the virus replication cycle is composed of many distinct stages, some viral proteins related to them, as the main-protease (Mpro) and RNA dependent RNA polymerase (RdRp), constitute individual potential antiviral targets. In this study, we challenged the mentioned enzymes against compounds pre-approved by health regulatory agencies in a virtual screening and later in Molecular Mechanics/Poisson–Bolzmann Surface Area (MM/PBSA) analysis. Our results showed that, among the identified potential drugs with anti-SARS-CoV-2 properties, Hypericin, an important component of the *Hypericum perforatum* that presents antiviral and antitumoral properties, binds with high affinity to viral Mpro and RdRp. Furthermore, we evaluated the activity of Hypericin anti-SARS-CoV-2 replication in an *in vitro* model of Vero-E6 infected cells. Therefore, we show that Hypericin inhibited viral replication in a dose dependent manner. Moreover, the cytotoxicity of the compound, in cultured cells, was evaluated, but no significant activity was found. Thus, the results observed in this study indicate that Hypericin is an excellent candidate for repurposing for the treatment of COVID-19, with possible inhibition of two important phases of virus maturation.

## Introduction

Since the coronavirus disease 2019 (COVID-19) pandemic was declared by WHO in March 2020, severe acute respiratory syndrome coronavirus 2 (SARS-CoV-2) has caused more than 260 million infections worldwide, with more than 5.1 million deaths (WHO, 2021). In Brazil, positive cases for COVID-19 have already reached more than 22 million and surpassed 600 thousand deaths (Ministério, 2021). Of note, the number of cases has experienced a decrease as the COVID-19 vaccines are being delivered worldwide. Despite that, the emergence of the virus variants,^1^ such as the alfa, gamma, delta, and the recently described omicron, in addition to the relaxation of pandemic restrictions has been associated with new waves of increasing number of cases regionally (Lemey et al., 2021; Naveca et al., 2021).

The clinical presentation of COVID-19 is characterized by the exhibition of distinct signs and symptoms, which influence the disease severity, ranging from asymptomatic and mild cases to acute respiratory distress syndrome (ARDS), respiratory and multiple organ failure, and ultimately death. Risk groups for the development of the severe COVID-19 comprise individuals of advanced age and who present some comorbidities, as pre-existing chronic medical conditions, such as diabetes (Zhou et al., 2020). A cytokine storm, associated with exacerbation of proinflammatory cytokine release due to the viral infection, is related to the emergence of ARDS and the evolution to the severe disease (Lucas et al., 2020; Ye et al., 2020).

Presently, the process of vaccination against SARS-CoV-2 is ongoing worldwide with distinct types of immunogens, including inactivated virus, adenoviral vectors, and viral RNA, among others (Abdulla et al., 2021; Kumar et al., 2021). Vaccines constitute one of the most important public health strategies to reduce disease burden. However, it is important to emphasize that there are important issues regarding access to vaccines globally, such as their uneven distribution and the need for differentiated infrastructure for their inter and intra-country dissemination, which compromises the coverage necessary for homogeneous immunological protection of populations (Günl et al., 2021). In addition, depending on the vaccine platform used, there are differences in their efficacy and safety in individuals from some risk groups and against the SARS-CoV-2 variants of concern (VOCs) (Harvey et al., 2021).

The therapeutic management of the infection with SARS-CoV-2 has changed significantly since the beginning of the pandemic. In Europe (European Medicines Agency^2^) and in the US (Food and Drug Administration^3^), so far, only the antiviral Remdesivir, a nucleoside analog that targets the viral RNA-dependent RNA polymerase (RdRp), and neutralizing antibodies have been approved as treatment options for COVID-19, in the modality of emergency use. However, their therapeutic benefits are still being fully determined. Additionally, several antiviral drugs have been investigated for the treatment of COVID-19 in clinical trials, such as Favipiravir, Lopinavir/Ritonavir, Umifenovir (arbidol), and the new drug Paxvolid (Jomah et al., 2020; Kumar et al., 2021; Mahase, 2021). Furthermore, host directed therapies, aiming to impair virus-host specific interface mechanisms, and immunomodulators that would counteract the exacerbated immune response associated with the disease severity are other relevant therapeutic options under investigation (Kumar et al., 2021).

Since the beginning of the COVID-19 pandemic, drug repurposing has been deployed as one agile mechanism for the identification of new SARS-CoV-2 targets for drugs already approved, however, outside the scope of its original nomination. Through this strategy, time and investment needed for drug development could be reduced as the greater part of the pre-clinical phase is already completed, especially the safety assessment phase and formulation development (Hernandez et al., 2017; Montes-Grajales et al., 2020; Egieyeh et al., 2021). In addition, repurposing decreases the chance that the compound will be unsuccessful in the clinical phases, as this step has usually been completed with the original indication (Ismail et al., 2021). Among one of the strategies used to identify active molecules is Structure-Based Virtual Screening (SBVS), a computational technique that uses the structural information of a protein from the pathogen to find possible inhibitors in a library of compounds that bind the protein with the highest affinity (Li and Shah, 2017). Usually, the classification of these compounds is done through molecular docking experiments, that is, the calculation of binding mode between the ligand and the receptor protein (Fradera and Babaoglu, 2018). Thus, docking added to the current computational power, and the use of virtual libraries of free compounds like ZINC15 (Sterling and Irwin, 2015) turn a personal computer into a powerful tool for drug search and design, which is highly advantageous for drug repurposing and also provides support for next steps of the drug development process (Montes-Grajales et al., 2020).

The virus replication cycle is composed of many distinct stages. Viral proteins acting in each of these stages each constitute individual potential antiviral targets. One of them is the SARS-CoV-2 main-protease (Mpro), also called 3-chymotrypsin-like protease (3CLpro), which is mainly responsible for processing the viral polyproteins (pp) 1a and 1ab into the non-structural proteins (NSPs), including the RNA-dependent RNA Polymerase (RdRp), the helicase, and the Mpro itself (Hegyi and Ziebuhr, 2002). Data from other studies have demonstrated that the activity of this enzyme is critical for replication of coronaviruses (Kim et al., 1995; Stobart et al., 2012). In addition, there are no described human analogs of this protein. Moreover, protease inhibitors are successfully implemented in the treatment against other viral diseases (Hoetelmans et al., 1997; Chary and Holodniy, 2010; Bacon et al., 2011). Altogether, these characteristics make the Mpro a promising antiviral drug target.

**FIGURE 1:**
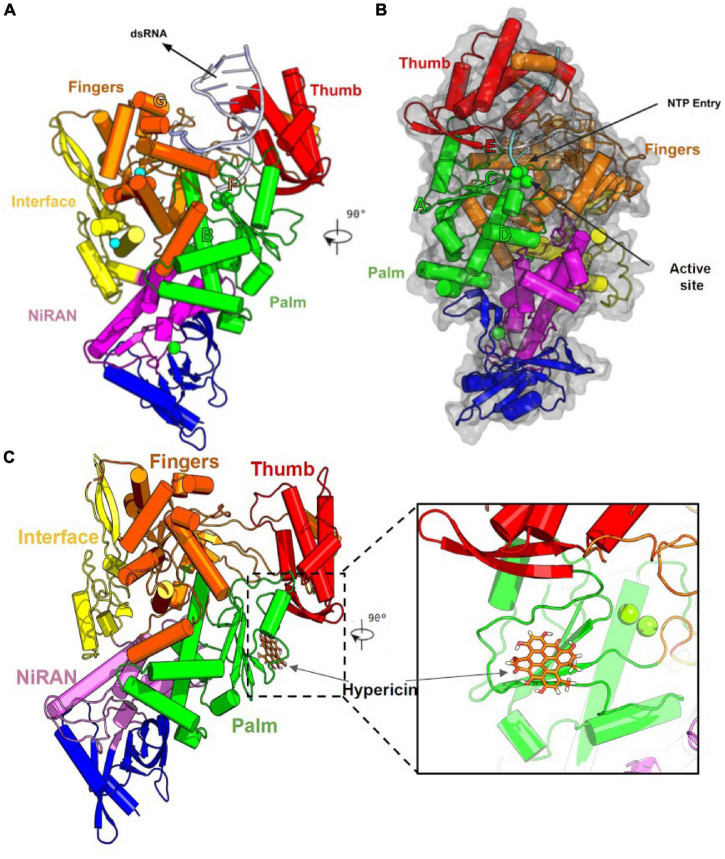
Structure of the SARS-CoV-2 RdRp polymerase in complex with Hypericin. **(A)** General structure of SARS-CoV2 RdRp and important parts of the active site. The Structure of SARS-CoV2 RdRp (NSP12) core contains an interface (Yellow), Nidovirus RdRp-associated nucleotidyltransferase (NiRAN in Magenta), and RdRp domains. The RdRp domain looks like a right hand containing the Thumb (in red), Palm (in green), and Finger (in orange) subdomains. **(B)** SARS-CoV2 presents a catalytic triad (GDD) at the center of the Palm subdomain that catalyzes the synthesis of an RNA strand complementary to the RNA template through the binding of NTP’s. Furthermore, the RdRp have six motifs named A to F that are well conserved. These motifs are responsible for template binding (Motif B), polymerization and recognition of NTPs (Motif D), coordination of ions and active site (motif A and C), conformational changes and support of the primer strand (Motif E). **(C)** Our docking calculations show that Hypericin interacts in the palm domain of RdRp near the catalytic center, represented by Mg ions. In this position, Hypericin blocks the natural pathway of ribonucleotides in the active site of the RdRp enzyme. Secondary structure of RpRd enzyme represented in cartoon style and colored according to protein domains, Mg ions represented in VDW and Hypericin is represented in orange licorice.

An additional relevant target is the RdRp, an enzyme that is responsible for the replication of viral RNA (Figure 1A), possessing an essential role in SARS-CoV-2 life cycle. This protein functions as a tripartite polymerase complex with NSP-7 and NSP-8 that further associates with NSP-14, which confers a proofreading exonuclease function (Subissi et al., 2014). Due to the particularities of this protein, several inhibitors for RdRp from Flaviviruses and SARS-CoV have been reported and some are being evaluated in pre-clinical and clinical phases (Niyomrattanakit et al., 2010; Montes-Grajales et al., 2020; Tian et al., 2021).

In this sense, the central goal of this work was to find compounds approved by international agencies as candidate therapeutic agents for repurposing against the SARS-CoV-2 through the SBVS experiment, specifically focusing on compounds that bind Mpro and RdRp. Secondly, we aimed to confirm the inhibitory action of promising compounds *in vitro*. As a result, one of the candidates identified was Hypericin, an antraquinone with antiviral and antifungal functions (Miskovsky, 2002). Additionally, we show that Hypericin acts as anti-SARS-CoV-2 replication inhibitor in μM concentrations in an *in vitro* model of Vero-E6 infected cells. These results qualify this drug as a promising antiviral candidate against SARS-Cov-2 and further experiments, including *in vivo* studies, are the next step of our experimentation.

## Methodology

### Structure Based Virtual Screening Experiment

In the SBVS experiment, essential proteins for the replication and maturation of SARS-CoV-2 were selected. The structures of the Mpro domain (pdb 6LU7) (Hatada et al., 2020) and the RdRp domain (7bv2) (Yin et al., 2021) from the Research Collaboratory for Structural Bioinformatics Protein Data Bank (RSCB PDB) (Berman et al., 2000) were used as receptor proteins. In both structures, all water and hetero-atoms were removed for docking experiments. In some experiments, Mg ions were maintained in the active site of the SARS-CoV-2 RdRp. Docking space was defined as a ±2 nm (in X, Y, Z) at the center of the active site. For the SBVS campaign, the enzymes Mpro and RdRp were challenged against ligand database (ZINC15/Enzyme/Trial and ZINC15/Enzyme/World) that have a total of 3,400 molecules already approved by several international regulatory agencies. HTP SurflexDock pipeline uses GROMACS 5.2 (Abraham et al., 2015) for molecular simulations and receptor ensemble sampling and Autodock 4.2 (Morris et al., 2009) for docking experiments (de Almeida Filho and Fernandez, 2020). In the initial HTP SurflexDock scoring results, a functional cut-off of Ki less than 10E-9 kcal/mol were considered as good candidates for re-scoring experiment. From this ranking, the 10 best inhibitors were evaluated for favorable binding on the protein active site and were re-evaluated through a post-processing step implemented in the HTP SurflexDock. Furthermore, the accurate binding energy inference (ΔΔG) of the most promising compounds was estimated using Molecular Mechanics/Poisson–Bolzmann Surface Area (MM/PBSA) methodology (Genheden and Ryde, 2015; Wang et al., 2018).

### The HTP SurflexDock Pipeline for Structure Based Virtual Screening

The HTP SurflexDock 1.0 pipeline^4^ is based on MDR SurflexDock pipeline (de Almeida Filho and Fernandez, 2020), modified to perform ‘docking and scoring’ experiments to classify promising compounds in SBVS experiments. Thus, we incorporated two types of post-processing analysis into the HTP SurflexDock pipeline: (1) Manual refinement and re-scoring of compounds and (2) Inference of binding free energy (ΔΔG) for most promising complexes using MM/PBSA. In the post-processing module for the inference of the ΔΔG calculated from molecular simulations based on the MM/PBSA protocol was used (Mobley and Dill, 2009; Genheden and Ryde, 2015). MM/PBSA is widely used in affinity inference analyses, as well as compound re-scoring (Wang et al., 2019). In this context, in HTP SurflexDock we used the g_mmbpsa software (Ren et al., 2020) for complex affinity inference. The g_mmpbsa is configured to calculate the free energy of the last 3 ns simulation and the initial 7 ns are used for the equilibrium of the system. At the end of the calculation, the python mmpbsa.py script is used to generate the graph of the ΔG variation as a function of time and a summary containing the averages of the energy contributions.

### Cell Culture

We used Vero E6 cells (African green monkey kidney cells) for SARS-CoV-2 isolation and propagation, as well as for assays of evaluation of the antiviral potential of the candidate compound. All cell culture reagents were from Gibco (Thermo Fisher Scientifc, Waltham, MA, United States). Sterile, pyrogen free, culture-treated plastic ware was purchased from Corning and Sarstedt. The basic culture medium used for Vero E6 cells consisted of Dulbecco’s Modified Eagle Medium (DMEM) formulated with D-glucose (4.5 g/l) and L-Glutamine (3.9 mM). Basic medium was supplemented with 100× penicillin-streptomycin solution (to final 100 U/ml and 100 μg/ml, respectively) and with inactivated fetal bovine serum (USDA-qualified region FBS) at 10%. Both cell and viral cultures were incubated at 37°C and 5% CO_2_.

### Severe Acute Respiratory Syndrome Coronavirus 2 Isolate

The SARS-CoV-2 isolate used in the assays was obtained from a respiratory sample collected from a COVID-19 patient diagnosed in March 2020, in Rio de Janeiro, Brazil. The original sample was a combination of two mid-turbinate nasal swabs and one pharyngeal swab, all collected in 3 ml of viral transport medium (DMEM supplemented with 1% bovine serum albumin and 1× penicillin-streptomycin). For virus isolation, 200 μl of the sample were inoculated in a confluent monolayer of Vero E6 cells in a T25 culture flask. Culture was incubated for 96 hs, with inspections for development of cytopathic effect and collection of supernatants every 24 h to evaluate viral replication. The viral isolate was further characterized by whole genome sequencing (published on gisaid.org, accession number EPI ISL 414045) and transmission electron microscopy (Barreto-Vieira et al., 2021). Viral titer of the isolate was increased by an additional passage in Vero E6 cells, to obtain a working stock. The 50% Tissue Culture Infectious Dose (TCID_50_) titer of the viral working stock was determined by limiting dilution and infection of Vero E6 cells. All the procedures related with the viral isolate culture and further treatment were performed in biosafety level 3 laboratory, in accordance with the WHO guidelines.^5^ Regarding ethical aspects, the patient sample used for viral isolation was collected at a sentinel health unit of the respiratory disease surveillance network of the Brazilian Ministry of Health, as part of routine procedures of the COVID-19 surveillance program. As the National Influenza Center and National SARS-CoV-2 Reference Laboratory for the surveillance network, our laboratory systematically receives respiratory samples for viral detection, sequencing, and isolation. All procedures involving patient samples were approved by the Committee of Ethics in Human Research of the Oswaldo Cruz Institute (registration number CAAE 68118417.6.0000.5248).

### Virus Inhibition Assay

All incubation steps of the assays were performed at 37°C and 5% CO_2_. First, cells were plated and cultured overnight to obtain confluent monolayers. Next day, cells were washed once with plain PBS, then, SARS-CoV-2 inoculums were incubated for one hour. The viral dose of the inoculums corresponded to a multiplicity of infection (MOI) of 0.01 TCID_50_. After infection, inoculums were removed from wells and replaced by the appropriate supplemented medium with distinct concentrations of the candidate compound. The candidate compound was diluted in NaOH (1 M), which was used as a control and was diluted similarly to the compound, reaching a concentration of 2 mM in the following experiments. Supernatants for viral quantification were collected 48 h post-infection (hpi).

### Viral Quantification

We evaluated SARS-CoV-2 replication in the candidate compound-treated versus non-treated cultures by measuring the number of viral RNA copies in the supernatants. For this purpose, we used the real time reverse transcription-polymerase chain reaction method (real time qRT-PCR) (Corman et al., 2020). This protocol employs TaqMan primers and probes specific to the gene encoding the envelope (E) protein. As quantification standard, we used a synthetic RNA molecule comprising the reference sequence of the E target, with a known number of copies (10^7^ copies/mL, kindly provided by Charité Virology through Pan American Health Organization). A concentration curve was prepared by serial dilution of the positive control from 10^6^ to 10 copies/mL. Viral RNA was extracted from 140 μL of cell-free culture supernatants using QIAamp Viral RNA mini kit (Qiagen, Hilden, Germany), according to manufacturer’s instructions. Reverse transcription and gene amplification were performed in one-step reactions with a qRT-PCR kit developed by the Biomanguinhos Institute (Fiocruz, Rio de Janeiro, Brazil), in ABI 7500 thermocycler (Applied Biosystems, Waltham, MA, United States). The candidate compound concentration required to decrease the viral RNA by 50% (*IC*_50_) was calculated using GraphPad Prism (GraphPad Software Inc., San Diego, CA, United States).

### Cellular Cytotoxicity

Vero E6 cells were plated at 10^4^ cells per well in 96-well plates and incubated overnight to obtain confluent monolayers. The following day, distinct concentrations of the candidate compound was added to the cultures, in triplicate. This was followed by incubation for 48 h at 37°C and 5% CO_2_. Cell supernatants were used to measure LDH released by cell death with the commercial kit CyQUANT*™* LDH Cytotoxicity Assay (Thermofisher, Waltham, MA, United States). Briefly, 50 μl of the supernatants from the treated cells and of the controls were transferred to a microtiter plate. Then, 50 μl of LDH colorimetric substrate was added to each sample and incubated for 30 min at room temperature, protected from light. LDH activity was determined by absorbance (OD) at 490/680 nm. Cytotoxicity was determined according to the manufacturer’s guidelines.

### Statistical Analysis

Statistical analysis was performed with GraphPad Prism (GraphPad Software Inc., San Diego, CA, United States) by using One-way ANOVA with Dunnett’s multiple comparison tests. Results were considered significant when *p* < 0.05.

## Results

### *In silico* Analysis of Main-Protease and RNA Dependent RNA Polymerase Inhibitor Candidates

To identify good candidates for repurposing for SARS-Cov-2 Mpro and RdRp, we made a SBVS experiment with these proteins and a set of 3,400 molecules using the HTP SurflexDock.

For SARS-Cov-2 Mpro, our analysis ranked nine promising compounds as nM and pM inhibitors: Nelfinavir, Hypericin, Mitoxantrone, Saquinavir, Remikiren, Aclarubicin, ZINC24447427, Indinavir, and Dihydroergotamine, which comprise a group of anti-HIV, antitumor, and antifungal drugs (Table 1).

**TABLE 1:** Docking hits for SARS-Cov-2 Mpro (pdb 6lu7 structure).

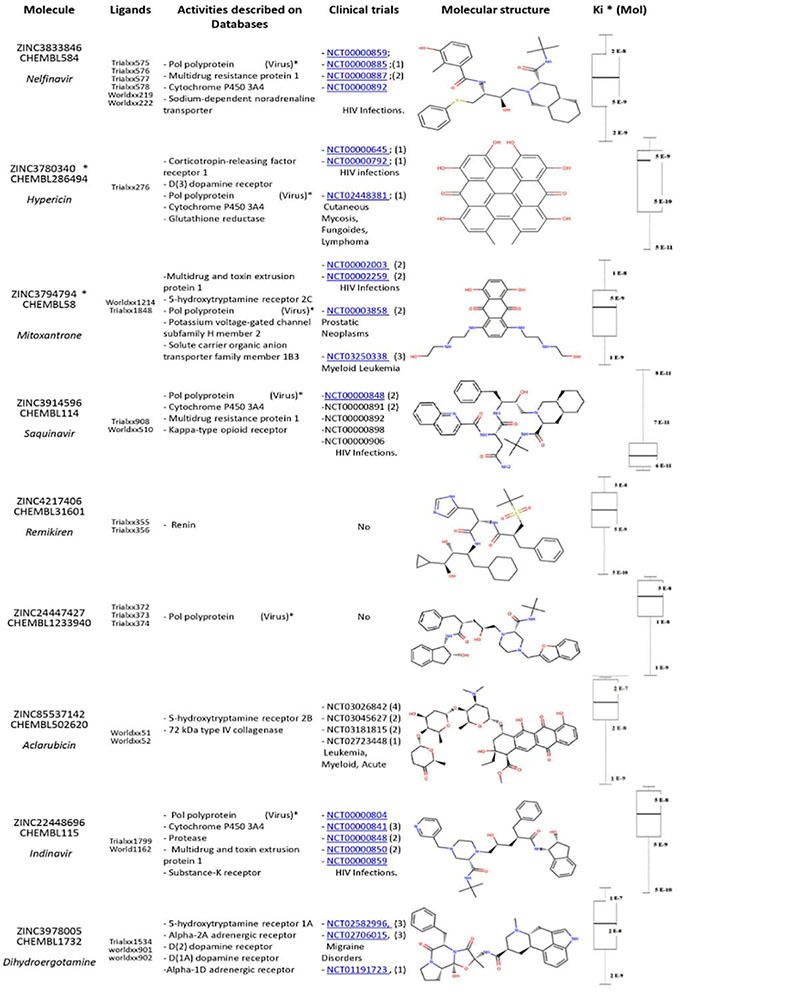

In addition, for SARS-Cov-2 RdRp our results indicated the 6 best candidates for RdRp inhibition as: Trypan blue, Hypericin, Mitoxantrone, Glycyrrhizinate Dipotassium, Lifitegrast, and Tudca, which obtained high affinity with the SARS-CoV-2 RdRp active site (Table 2) and represent molecules in clinical testing phases for treatment of neoplasms, lymphomas, antifungals, anti-HIV among other applications, which may indicate that these compounds are good candidates for broad-spectrum therapeutic antivirals.

**TABLE 2:** Docking hits for SARS-Cov-2 RdRp (pdb 7aap structure).

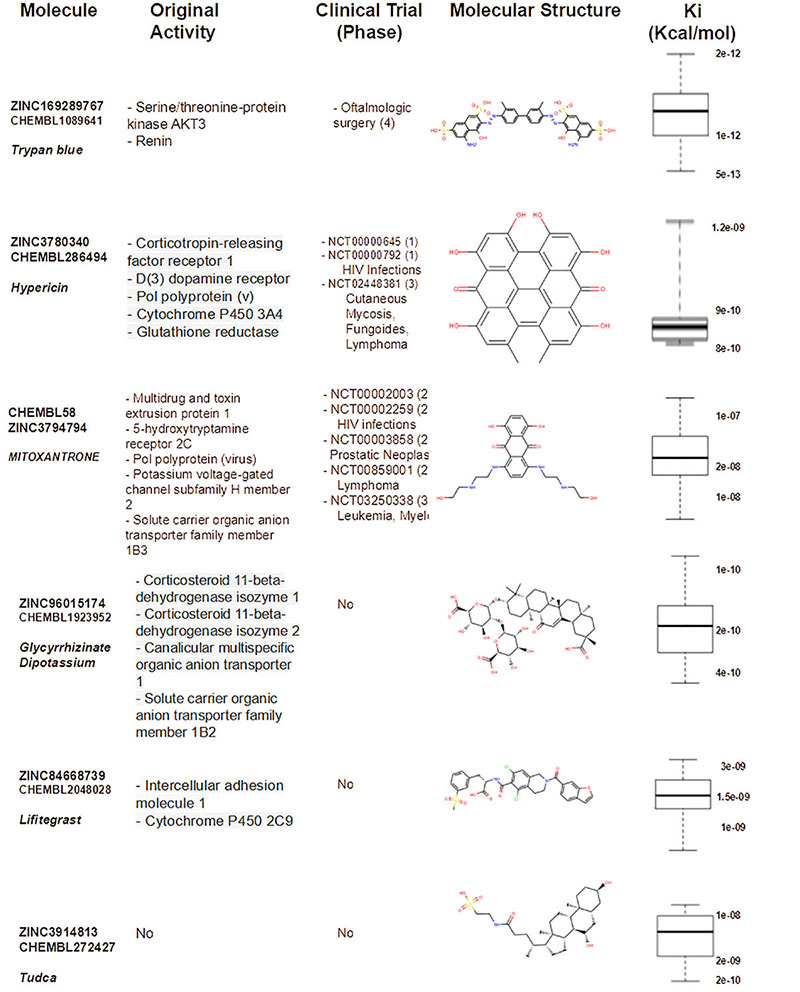

Obtained results suggest Hypericin and Mitoxantrone as best candidates for *in vitro* further experiments, as pointed as nM inhibitor for both enzymes in docking experiments. Moreover, for RdRp-Hypericin complex, a consecutive MM/PBSA experiment pointed a stable interaction of Hypericin near motif C in the active site (Figures 1B,C) with calculated Δ*G* = −22.704 ± 4.008 Kcal/mol (Supplementary Figure 1). Most of the other well-ranked molecules for the RdRp enzyme in our *in silico* experiments also are potential candidates for repurposing for the therapeutic treatment of SARS-CoV2.

### Hypericin Reduces Replication of Severe Acute Respiratory Syndrome Coronavirus 2 *in vitro* at Non-cytotoxic Concentrations

**FIGURE 2:**
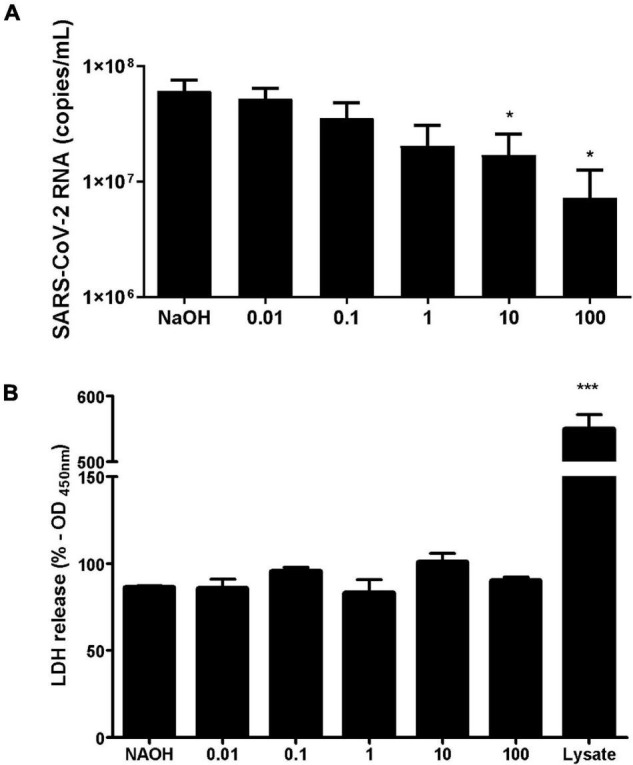
Hypericin inhibits replication of SARS-CoV-2 *in vitro* at non-cytotoxic concentrations. **(A)** Inhibition of SARS-CoV-2 replication by hypericin. Vero E6 cells were infected with SARS-CoV-2 for 1 h at a MOI of 0.01. Infected cells were then treated with increasing concentrations of hypericin (0.01–100 μM) for 48 hpi or NaOH (2 mM) as vehicle control. Viral RNA was quantified from the collected supernatants at 48 hpi by real time qRT-PCR. Bars show mean and SEM of four independent experiments. One-way ANOVA with Dunnett’s multiple comparison tests, *p < 0.05. **(B)** Cytotoxicity of hypericin in Vero E6 cell line. Presence of LDH was measured in the supernatant of cells treated with the indicated concentrations of the compound for 48 h. Cell lysate was used as a positive control. Bars show mean and SEM of three independent experiments. One-way ANOVA with Dunnett’s multiple comparison tests, ***p < 0.0001.

As the *in silico* analysis demonstrated that Hypericin was a potential candidate for the binding and inhibition of SARS-CoV-2 Mpro and RdRp proteins, we were interested in analyzing if this potential interaction would have an impact on the replication of the SARS-CoV-2 in our model of *in vitro* infection with the virus. Our results show that Hypericin significantly reduced viral replication in a concentration dependent fashion (Figure 2A). The highest Hypericin concentrations tested (10 and 100 μM) resulted in the highest degree of reduction in supernatant viral RNA (*p* < 0.05) and reached 84 and 96% of inhibition, respectively.

Moreover, to confirm that the reduction in viral replication was related to the inhibition of the viral replication cycle and not to an indirect effect on the host cell, we next evaluated the potential cytotoxic effects of the compound by measuring the release of LDH in non-infected cells for 48 h. As a result, there was no significant increase in extracellular LDH in cells treated with increasing concentration of the drug (0.01–100 μM), as compared to the cells treated with the drug vehicle NaOH (Figure 2B). Of note, total cell lysate was used as a positive control and presented a significant increase in LDH detection. These findings strengthen our computational screening experiments as Hypericin has come out to be one of the most interesting hits against the two viral proteins.

## Discussion

Almost 2 years after the identification of SARS-CoV-2 and the declaration of the COVID-19 pandemic, many treatment options have been investigated, but just a few have displayed enough effect against the disease to be considered for emergency use, such as remdesivir and monoclonal antibodies, or to be regarded as a promising therapy by the health authorities, such as Paxlovid.^6^

Furthermore, they are targeted to specific clinical stages of the disease or patient groups. In search for more therapeutic options to contain the morbidity and mortality caused by SARS-CoV-2, drugs that target the viral proteins and the host molecules that drive the response against infection are being studied, as combination therapies are a good approach to successfully fight this disease (Kumar et al., 2021). Also, drug repurposing has been highlighted as a relevant strategy to speed up the identification of compounds with anti-SARS-CoV-2 activity and, more importantly, reduce time and cost for clinical implementation of any potential drugs identified in experimental conditions.

Here, we screened a database of 3,400 known chemical compounds by computational analysis, to identify molecules able to interact with the viral proteins Mpro and RdRp, both essential for replication of SARS-CoV-2. The ligands were further classified according to their affinity of interaction with the viral targets, by molecular docking. This analysis indicated several compounds with high affinity to the viral proteins that could, in theory, display anti-SARS-CoV-2 properties. In general, viral protease inhibitors are widely studied drugs by the academic community. Many of ranked compounds identified in this study include inhibitors developed for HIV treatment (Bardsley-Elliot and Plosker, 2000), most of them (Indinavir, Saquinavir, Indinavir and Hypericin) already tested *in silico* or *in vitro* as candidates for repurposing for the SARS-CoV-2 (Bello et al., 2020). Obtained here results for SARS-Cov-2 Mpro (Table 1) were in concordance with the general picture found in other *in silico* SBVS academic works and were interpreted as excellent positive control for our SBVS experimental pipeline. Interestingly, Hypericin appeared as one of the top hits in the panel of possible ligands for both Mpro and RdRp viral proteins (Tables 1, 2). Moreover, we showed that Hypericin reduced SARS-CoV-2 replication at μM concentrations in an *in vitro* experimental model and had no significant cytotoxic effect in the same model (Figures 1, 2).

Hypericin is an anthraquinone member of the naftodianthrone class of chemical components obtained primarily from plants of the genus *Hypericum*, particularly *Hypericum perforatum* (commonly known as St. John’s wort). Previous reports have shown that Hypericin presents antiviral activity against some viruses, such as hepatitis C, HIV, and influenza A (Lenard et al., 1993; Jacobson et al., 2001; Pu et al., 2009; Shih et al., 2018), as well as for the avian coronavirus IBV (Chen et al., 2019). Additionally, Hypericin possess antitumor properties (Rook et al., 2010; Dong et al., 2021). Recent studies have reported relevant interaction between Hypericin and Mpro by molecular docking analysis with *IC*_50_ of 65 μM (Pitsillou et al., 2020a,b; Shivanika et al., 2020; Yalçın et al., 2021) and by inhibition of the protease activity *in vitro* (Pitsillou et al., 2020a), although in a different report employing fluorescence resonance energy transfer (FRET) experiments, Hypericin was considered a weak SARS-Cov2 Mpro inhibitor (Loschwitz et al., 2021). In addition, some studies evaluated *in silico* the interaction of Hypericin with the SARS-CoV-2 Spike, papain-like protease (PLpro) and NSP14 proteins (Pitsillou et al., 2020b,^2021^; Romeo et al., 2020; Liu et al., 2021). However, none of these previous reports have demonstrated Hypericin antiviral activity against SARS-CoV-2 isolates.

Noteworthy, among additional antivirals identified, ranked compounds include inhibitors developed during the 1990s for HIV treatment (Noble and Faulds, 1996; Plosker and Noble, 1999; Bardsley-Elliot and Plosker, 2000). These are widely studied drugs by the academic community and has already been explored as possible candidates for repurposing for the SARS-CoV2 by other authors (Bello et al., 2020). In this sense, Ohashi and co-workers point out that Nefilnavir can block the replication of SARS-CoV-2 in synergy with the anti-inflammatory Cefarantin *in vitro* (Ohashi et al., 2021). In addition, *in silico* simulations performed by Yamamoto et al. (2020) indicate that Nelfinavir inhibits virus replication by binding to the Mpro protein, while Cefarantin interferes with the interaction of Spike protein with the ACE2 enzyme, preventing intrusion into the cell. Other studies indicate that Nelfinavir also has a systemic effect by avoiding the oxytocin storm in patients infected with HIV-1 and, in this context, Xu et al. (2020) suggest that Nelfinavir can prevent complications caused in severe cases of COVID-19. Another well-rated antiretroviral in our tests was Saquinavir, widely used in the treatment of SARS together with Ribavirin in the 2003 epidemic (Tan et al., 2004). A study by Yamamoto et al. (2020) pointed out that Saquinavir can inhibit SARS-CoV2 replication at low concentrations (*EC*_50_ = 8.83 μM) and suggests that the drug prevents the entry of the virus into the cell in addition to inhibiting the viral replication. Finally, Indinavir is also an antiretroviral that has also been well ranked in other SBVS but without *in vitro* validation (Shah et al., 2020). In the antitumor drug group, Mitoxantrone is an agent of the anthraquinone family used in the treatment of leukemia (Lokhande et al., 2021). *In silico* simulations with the Mpro of SARS-CoV2 indicate that Mitoxantrone binds strongly to the active site of enzyme through a network of hydrogen bonds and hydrophobic interactions inhibiting the replication of the virus (Farag et al., 2021). On the other hand, Aclarubicin is a drug of the anthracycline family in clinical trials for the treatment of acute myeloid leukemia. This drug has been evaluated *in silico* as a possible inhibitor of the interaction RBD-ACE2, hampering virus entrance into the host cell (Senathilake et al., 2020).

In conclusion, most of the well-ranked molecules for the Mpro and RdRp enzymes in our *in silico* experiments are good candidates for repurposing for the therapeutic treatment of SARS-CoV-2 and, among them, Hypericin presented promising results as a drug not previously evaluated for antiviral activity against SARS-CoV-2. Further experiments are under way, such as definition of Hypericin anti-SARS-CoV-2 *IC*_50_ in human cellular *in vitro* model by determination of specific virus viability after hypericin treatments and confirmation of Hypericin specific antiviral mechanisms of action and to compare its activity to other approved drugs like Remdesivir. Also in the scope of our interest is a phase I dose escalation study to determine antiviral activity of hypericin against SARS-Cov-2 and the safety in animal models.

## Data Availability Statement

The original contributions presented in the study are included in the article/Supplementary Material, further inquiries can be directed to the corresponding author/s.

## Author Contributions

ARM, BCC, JLAF, MMS, and JHF designed the experiments and wrote the manuscript. ARM, BCC, JLAF, JSCCM, MGPO, and TCS conducted the experiments. MAPH provided the access and the training for working in BSL-3 facilities that are necessary to perform all experiments involving live SARS-CoV-2 virus manipulation. All authors contributed to manuscript formulation and review and approved the submitted version.

## Conflict of Interest

The authors declare that the research was conducted in the absence of any commercial or financial relationships that could be construed as a potential conflict of interest.

## Publisher’s Note

All claims expressed in this article are solely those of the authors and do not necessarily represent those of their affiliated organizations, or those of the publisher, the editors and the reviewers. Any product that may be evaluated in this article, or claim that may be made by its manufacturer, is not guaranteed or endorsed by the publisher.

## Footnotes

1
who.int/en/activities/tracking-SARS-CoV-2-variants

2
ema.europa.eu

3
fda.gov

4
https://htpsurflexdock.biocomp.uenf.br/

5
who.int/publications/i/item/WHO-WPE-GIH-2021.1

6
www.pfizer.com/news/press-release/press-release-detail/pfizers-novel-covid-19-oral-antiviral-treatment-candidate

## Supplementary Material

Supplementary Figure 1 Molecular Mechanics/Poisson–Bolzmann Surface Area experimentation pointed to a stable interaction in Hypericin-SARS-Cov-2 RdRp complex. **(A)** Best docking results suggest that Hypericin interacts with RdRp active site near motif C and the Mg ion coordination residues, thus acting as competitive inhibitor of the ribonucleotide in RdRp polymerase activity. Residues interacting with Hypericin were numbered according to the nomenclature of 7bv2 pdb (Yin et al., 2021). **(B)** Calculated Δ*G* = −22.704 ± 4.008 Kcal/mol over the 60 ns of complex simulation. After small fluctuation, complex interaction was stable over the last 20 ns of simulation. **(C)** Energetic contribution of residues contacting the RdRp catalytic site in Hypericin binding, according to color code mapped on the secondary structure os the SARS-Cov-2 RdRp. On average, residues in blue contributed negatively to the interaction with Hypericin while residues in green, yellow and red favored the binding (in Kcal/Mol) over the 60 ns of simulations. Energetic contributions were calculated according to the methodology in (g_mmpbsa/single_protein_ligand_energy_contributions.html), thus modified for using Pymol 1.8.6 (www.pymol.org) for the visualization of the structures.
