# Supplementary figures and images for: Identification of Hypericin as a Candidate Repurposed Therapeutic Agent for COVID-19 and Its Potential Anti-SARS-CoV-2 Activity
Source: Front Microbiol. 2022 Feb 10;13:828984. doi: 10.3389/fmicb.2022.828984 (PMC8866965; doi:10.3389/fmicb.2022.828984)

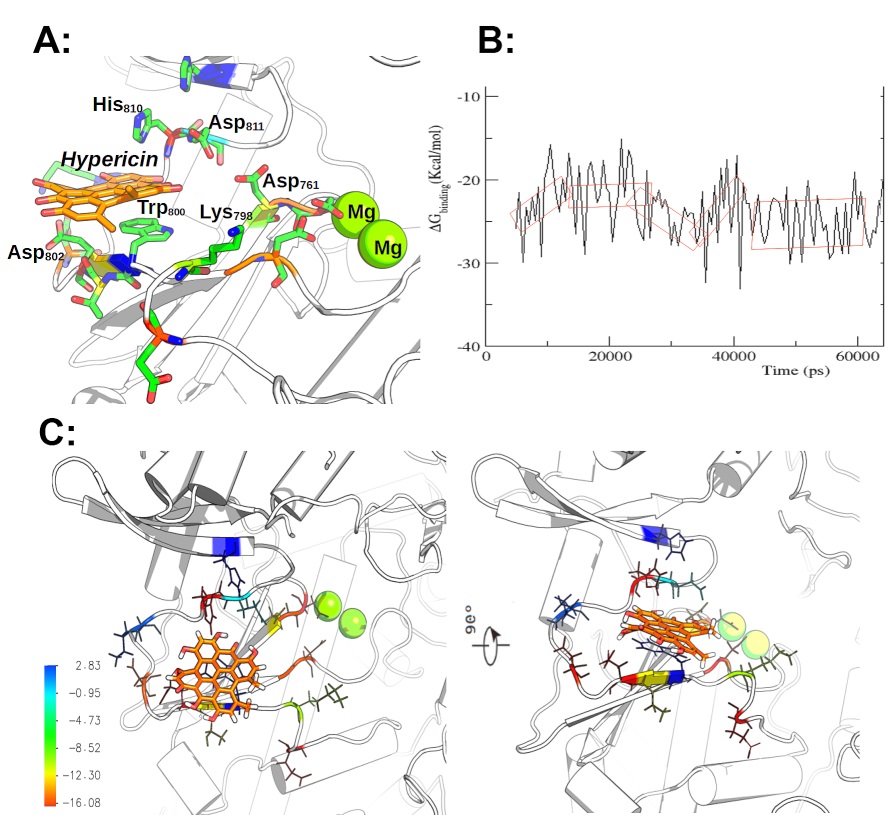

Supplement: Supplementary Figure 1 — Molecular Mechanics/Poisson–Bolzmann Surface Area experimentation pointed to a stable interaction in Hypericin-SARS-Cov-2 RdRp complex. (A) Best docking results suggest that Hypericin interacts with RdRp active site near motif C and the Mg ion coordination residues, thus acting as competitive inhibitor of the ribonucleotide in RdRp polymerase activity. Residues interacting with Hypericin were numbered according to the nomenclature of 7bv2 pdb (Yin et al., 2021). (B) Calculated ΔG = −22.704 ± 4.008 Kcal/mol over the 60 ns of complex simulation. After small fluctuation, complex interaction was stable over the last 20 ns of simulation. (C) Energetic contribution of residues contacting the RdRp catalytic site in Hypericin binding, according to color code mapped on the secondary structure os the SARS-Cov-2 RdRp. On average, residues in blue contributed negatively to the interaction with Hypericin while residues in green, yellow and red favored the binding (in Kcal/Mol) over the 60 ns of simulations. Energetic contributions were calculated according to the methodology in (g_mmpbsa/single_protein_ligand_energy_contributions.html), thus modified for using Pymol 1.8.6 (www.pymol.org) for the visualization of the structures. [file Image_1.JPEG]
